# Trustworthy Agentic AI in Bioinformatics: From Workflow Automation to Traceable and Validated Biological Inference

**DOI:** 10.3390/biology15171537

**Published:** 2026-09-04

**Authors:** Mia Yang Ang, Leonard Lipovich, Siew Woh Choo, Li Chen, Lanni Song

**Affiliations:** 1 Department of Biomedical Sciences, Jeffrey Cheah Sunway Medical School, Faculty of Medical and Life Sciences, Sunway University, Sunway City, Petaling Jaya 47500, Selangor, Malaysia; 2 Sunway Microbiome Centre, Faculty of Medical and Life Sciences, Sunway University, Sunway City, Petaling Jaya 47500, Selangor, Malaysia; 3 Zhejiang-Malaysia Joint Laboratory for Rare Medicinal Resources, Wenzhou-Kean University, 88 Daxue Road, Ouhai, Wenzhou 325060, China; 4 College of Science, Mathematics and Technology, Wenzhou-Kean University, 88 Daxue Road, Ouhai, Wenzhou 325060, China; 5 Dorothy and George Hennings College of Science, Mathematics and Technology, Kean University, 1000 Morris Ave, Union, NJ 07083, USA; 6 International Frontier Interdisciplinary Research Institute (IFIRI), Wenzhou-Kean University, 88 Daxue Road, Ouhai, Wenzhou 325060, China

**Keywords:** agentic artificial intelligence, bioinformatics, computational biology, omics analysis, scientific provenance, epistemic traceability, biological validation, reproducibility, human oversight, accountable autonomy

## Abstract

Agentic artificial intelligence is extending bioinformatics beyond conversational assistance by enabling systems to select tools, execute code, revise analytical plans, and interpret biological data. These capabilities may accelerate research, but they also redistribute decisions that determine whether biological conclusions are valid. We conducted a targeted, structured PubMed search in July 2026 and identified 11 peer-reviewed agentic bioinformatics systems for descriptive review based on predefined eligibility criteria for analytical decision-making, tool or code execution, iterative evaluation, or coordinated agent activity. The evidence base covered single-cell transcriptomics, microbial genomics, cancer genomics, and omics applications, together with methodological literature on reproducibility and biological validation. We examined how current systems report delegated authority, provenance, validation, evidence, abstention, and human oversight. Existing platforms implement safeguards such as sandboxed execution, restricted commands, interaction logs, evidence identifiers, automated checks, critic agents, quality scores, and expert assessment. However, published reports rarely provide a connected account linking the original biological question to samples, reference resources, analytical decisions, computational actions, statistical results, supporting evidence, validation outcomes, and final claims. We distinguish inherited bioinformatics errors, errors amplified through autonomous action, and emergent failures arising from memory, retrieval, tool interaction, or agent coordination. We further propose a multidimensional decision-rights profile, consequence-sensitive validation gates, and a claim-to-evidence provenance architecture organized through the Traceable History of Research Evidence, Agent Actions, and Decisions in Bioinformatics (THREAD-Bio) framework. Illustrative cases show that technically successful execution may still support misleading inference. Trustworthy agentic bioinformatics therefore requires claims to remain reconstructible, challengeable, validated, and proportionate to the evidence.

## Simple Summary

Artificial intelligence agents are beginning to assist researchers with bioinformatics tasks, including analysing genes, cells, microbes, and disease-related data. These systems can select software, run commands, inspect results, and revise analytical plans. Although this may save time, a completed workflow can still produce an unreliable biological conclusion. Problems may arise when an agent uses unsuitable samples, applies the wrong reference genome, overlooks the study design, chooses inappropriate statistics, or interprets results more confidently than the evidence allows. This review explains how such errors can originate from ordinary bioinformatics practice, become amplified through autonomous actions, or emerge from agent-specific features such as memory, retrieval, and tool coordination. It also examines safeguards already used in published systems, including restricted computing environments, activity records, automated checks, evidence links, and expert review. We propose that trustworthy agent-assisted research should preserve a connection between the original biological question, the data used, the analytical choices made, the results produced, the evidence supporting each claim, and the people responsible for approval. Examples from single-cell analysis, microbial genomics, and cancer genomics show why technically successful analyses may still be biologically misleading. Better traceability can help researchers identify weak claims, stop unsafe analyses, and improve transparency, reproducibility, and accountability.

## 1. Introduction: From Workflow Automation to Accountable Biological Inference

Bioinformatics has long combined computational automation with expert scientific judgement. Conventional pipelines organize predefined operations for quality control, alignment, quantification, statistical analysis, and biological interpretation. Workflow-management systems further improve portability and reproducibility across computing environments. Nevertheless, successful execution does not ensure that an analysis is scientifically valid. Researchers must still determine whether the selected samples address the intended question, whether reference resources are compatible with the data, whether statistical models represent the experimental design, and whether the resulting conclusions are proportional to the available evidence.

These challenges become more pronounced as studies integrate genomic, transcriptomic, proteomic, metabolomic, imaging, and clinical data. Multi-omics integration can reveal relationships that are not apparent from individual data types, but it also introduces heterogeneity in measurement scales, missingness, data quality, and inferential assumptions [1,2,3,4,5]. Clinical next-generation sequencing workflows face additional difficulties involving laboratory variation, reference selection, data processing, and interpretation [6]. Similar reproducibility challenges occur in conservation genomics and other rapidly developing biological disciplines [7]. Precision-oncology analyses must further distinguish technical detection from clinical interpretation and actionability [8]. Single-cell studies require careful treatment of biological replication, batch effects, cellular heterogeneity, and cell-state annotation [9,10].

Agentic artificial intelligence introduces an additional layer of complexity because these systems can participate directly in analytical decision-making. Rather than only responding to questions or generating isolated code, an agent may interpret a research objective, select tools, execute commands, inspect intermediate outputs, revise its plan, and produce biological explanations. Recent reviews have described agent architectures, foundation models, applications, hallucination, reproducibility, and governance in automated bioinformatics [11,12]. Reliability, reproducibility, and risk-aware interpretation have also been emphasized for large-language-model use in microbial genomics [13]. These reviews establish the broader landscape of AI-assisted bioinformatics, but they do not jointly examine which scientific decisions are delegated to agents, how consequential decisions are validated, and whether individual biological claims can be traced back to the data, analyses, evidence, and approvals that support them. The present review addresses this gap by considering delegated scientific authority, validation, provenance, and claim-level accountability as connected components of the same inferential process.

This emphasis on accountability also reflects broader principles of AI governance. The OECD AI Principles emphasize transparency and explainability, robustness and safety, and accountability in the development and use of trustworthy AI [14]. In agentic bioinformatics, these principles have direct scientific implications: researchers need to know what an agent was permitted to decide, what evidence supported consequential actions, and where responsibility for approving biological conclusions remained with human investigators.

### 1.1. From Fixed Workflows to Decision-Making Bioinformatics Agents

A conventional workflow follows a predefined sequence of operations, although researchers may still make important decisions before and after execution. An agentic system can introduce additional decisions while the analysis is running. It may substitute a tool after an installation failure, modify a threshold when an output is empty, retrieve updated documentation, revise the analytical objective, or prioritize a result that appears biologically coherent. These capabilities may improve flexibility and efficiency, but they also expand the range of analytical decisions that a system can influence. Technical capability must therefore be distinguished from delegated scientific authority. A system may be able to change a statistical model or sample filter without being authorized to alter the primary analysis.

AutoBA [15] performs analytical planning, code generation, execution, and automated repair across multiple omics tasks. BRAD [16] connects language-model planning with literature retrieval, biological databases, and analytical modules for biomarker discovery. BioAgents [17] provides conceptual genomics support and workflow recommendations. BioMedAgent [18] coordinates tools across cross-omics, machine-learning, and biomedical-imaging analyses. Agentomics [19] conducts iterative biomedical machine-learning experimentation under programmatic checkpoints. Related AI-assisted systems such as IAN [20] also support omics analysis and data-driven discovery. These systems differ in scope and architecture, but each can influence decisions that would traditionally be made or reviewed by a bioinformatician.

Other systems are designed for specific biological domains. CellVoyager [1] performs notebook-based exploration of single-cell RNA-sequencing data and uses intermediate findings to guide subsequent analyses. CASSIA [21] distributes cell-type annotation across annotator, validator, scorer, and reporter agents. BIOGEN [22] combines evidence retrieval with multi-critic interpretation of bacterial transcriptomic clusters associated with antimicrobial resistance. Viral Sentry AI [23] links viral-genome surveillance with host-risk prediction and downstream drug-repurposing analysis. AI-HOPE-WNT [24] supports conversational analysis of WNT-pathway dysregulation in colorectal cancer. AI-HOPE-PM [25] integrates genomic, clinical, and social variables in precision-medicine analyses. Related applications extend to spatial omics [26], plant biology [27], and multi-agent scientific discovery [11]. Because these systems address different biological questions, their outputs cannot be evaluated using a single generic measure of task completion.

### 1.2. From Technical Completion to Accountable Biological Inference

Successful execution establishes only that a computational operation produced an output. It does not establish that the operation was appropriate for the study design or that the resulting biological interpretation is defensible. A differential-expression analysis may run correctly even when individual cells are incorrectly treated as independent biological replicates. A survival model may converge after subgroups have been selected by examining the same outcome data. A pathway-enrichment tool may return statistically significant terms when the background gene universe is inappropriate. A phylogenetic workflow may be reproducible while supporting an unjustified claim of direct transmission. These are established bioinformatics problems rather than failures created exclusively by artificial intelligence.

The distinctive concern is that an agent may connect planning, execution, interpretation, and reporting within the same iterative process. An incorrect decision made early in the workflow may therefore propagate through several downstream stages without independent review. Automated repair is useful when it corrects a malformed command, missing dependency, or incompatible file path. However, a retry becomes scientifically consequential when it changes a quality-control threshold, genome build, annotation resource, clustering resolution, statistical contrast, or primary estimand. If only the final successful run is retained, the reported result may conceal the analytical alternatives that produced it. Additional risks may arise from persistent memory, external retrieval, tool permissions, and instructions embedded in accessible resources [28].

**Figure 1:**
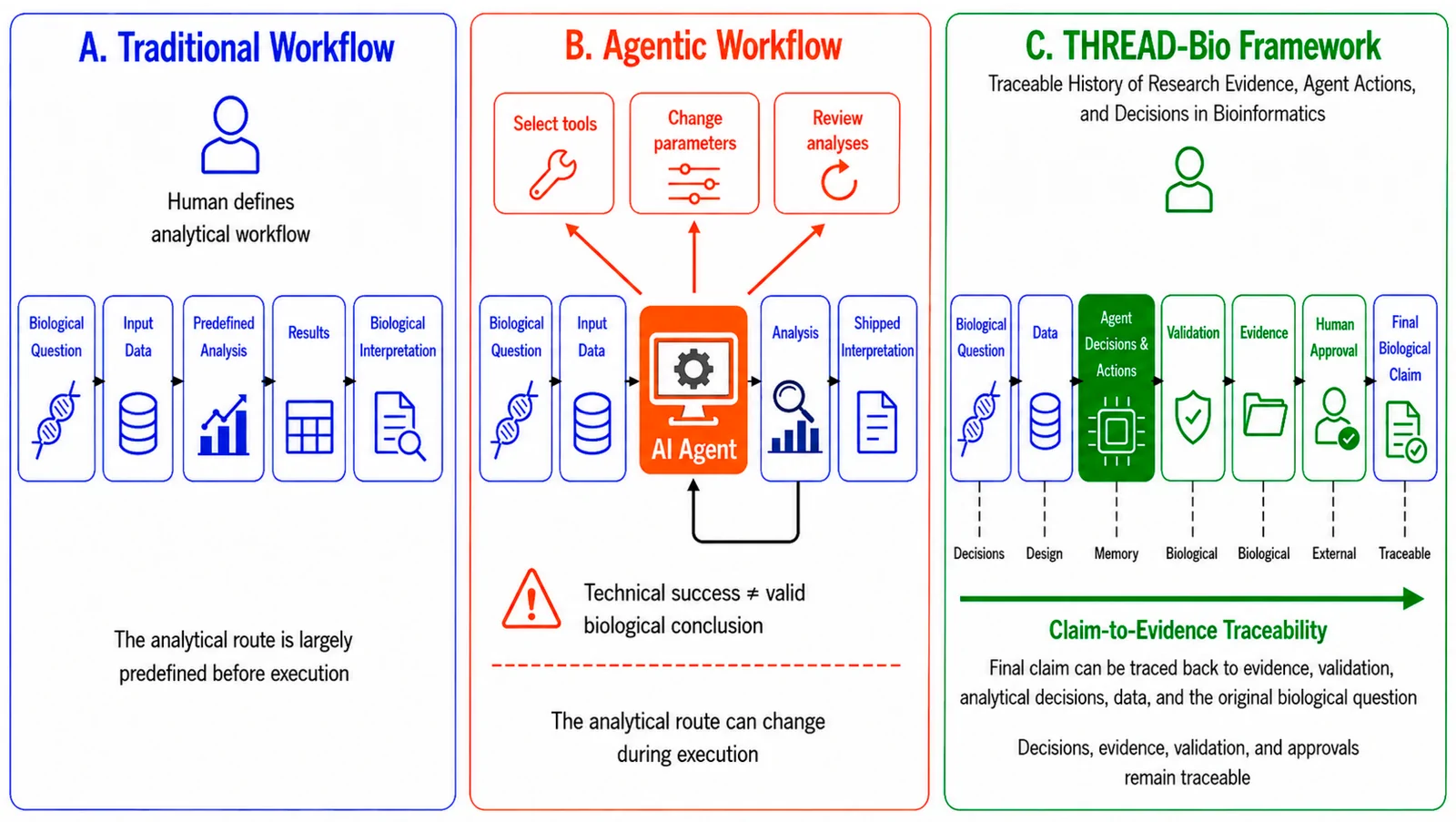
Progression from traditional bioinformatics workflows to agentic analysis and the proposed THREAD-Bio framework. (**A**) Traditional workflows follow a largely predefined analytical path. (**B**) Agentic workflows allow AI agents to adapt tools, parameters, and analyses during execution. (**C**) THREAD-Bio adds validation, evidence tracking, human oversight, and claim-to-evidence traceability to support accountable biological inference.

This review addresses these concerns through four connected contributions. First, it distinguishes conventional bioinformatics failures from errors amplified through autonomous action and failures arising from agent-specific mechanisms. Second, it introduces a conceptual decision-rights profile covering data, methods, parameters, statistics, execution, interpretation, and reporting. Third, it proposes consequence-sensitive validation gates and a claim-to-evidence provenance architecture linking biological claims to the data, decisions, computations, and evidence from which they were derived. Finally, it presents the Traceable History of Research Evidence, Agent Actions, and Decisions in Bioinformatics, or THREAD-Bio, as a proposed reporting framework. THREAD-Bio is not proposed as an established minimum-information standard. It is intended as a structured approach for making agent-assisted biological inference reconstructible, challengeable, appropriately validated, and accountable under human oversight. In practical terms, THREAD-Bio connects the original biological question and study design with data and reference provenance, agent actions and analytical decisions, computational outputs, validation results, supporting or contradictory evidence, human approvals, and the final biological claim. It is intended to preserve an inspectable route from question to claim without requiring disclosure of private model reasoning. The transition from a fixed bioinformatics workflow to agentic decision-making and, subsequently, to THREAD-Bio-based accountable inference is summarized in Figure 1.

## 2. Literature Search and Review Methodology

### 2.1. Search Strategy and Eligibility

A targeted, structured PubMed search was conducted in July 2026 to identify literature on agentic artificial intelligence in bioinformatics and methodological studies relevant to reliability, reproducibility, validation, provenance, and biological interpretation. Twenty-two targeted query groups covered agentic bioinformatics systems and related topics, including single-cell analysis, tool and code execution, statistical validity, biological validation, microbial and cancer genomics, human oversight, reporting, and benchmarking. The principal system-identification query was:

((“agentic AI”[Title/Abstract] OR “AI agent”[Title/Abstract] OR “AI agents”[Title/Abstract] OR “artificial intelligence agent”[Title/Abstract] OR “artificial intelligence agents”[Title/Abstract] OR “LLM agent”[Title/Abstract] OR “LLM agents”[Title/Abstract] OR “autonomous agent”[Title/Abstract] OR “autonomous agents”[Title/Abstract] OR “multi-agent”[Title/Abstract] OR “multi-agent system”[Title/Abstract] OR “multi-agent systems”[Title/Abstract]) AND (bioinformatics[Title/Abstract] OR genomics[Title/Abstract] OR omics[Title/Abstract] OR transcriptomics[Title/Abstract] OR proteomics[Title/Abstract] OR metabolomics[Title/Abstract] OR “computational biology”[Title/Abstract])) NOT preprint[Publication Type]

Relevant PubMed-indexed publications already known to the authors and additional studies identified during claim-level citation checking were also considered. Preprints were excluded; where both a preprint and peer-reviewed journal version were available, only the journal publication was retained.

For the focused review of agentic systems, eligible studies described an implemented system applied to bioinformatics, computational biology, genomics, transcriptomics, proteomics, metabolomics, microbiome research, or related omics applications and demonstrated substantive involvement in the analytical process. A system was considered agentic when it could determine or revise at least part of the analytical route between a research objective and its output, including through planning or replanning, tool selection or execution, adaptation to intermediate results, or coordination among specialized agents. Conventional deterministic workflows with largely predefined analytical sequences, and conversational systems limited to explanation, isolated code generation, or question answering, were excluded.

Methodological and contextual studies were included separately when they directly informed the review’s analysis of scientific validity, reproducibility, provenance, validation, biological interpretation, human oversight, or reporting. Duplicate records and publications outside the scope of the review were excluded.

PubMed was selected to provide a defined biomedical literature boundary. This approach may omit relevant developments reported primarily in computer-science conference proceedings, preprint repositories, software repositories, or non-PubMed databases. Accordingly, this article is intended as a targeted critical review rather than an exhaustive or systematic census of agentic systems in computational science.

### 2.2. Literature Synthesis and Descriptive Review

The included literature was reviewed narratively to examine how agentic artificial intelligence is currently being applied in bioinformatics and what scientific issues arise when analytical decisions are increasingly delegated to these systems. For publications describing implemented systems, descriptive information was considered on their biological application, data type, general agent architecture, analytical functions, reported validation approaches, provenance mechanisms, and involvement of human researchers. Information was included only when it was explicitly described in the publication or associated supporting material. Features that were not sufficiently described were treated as not reported rather than assumed to be absent.

The purpose of this review was not to rank, score, or benchmark individual systems. The published systems instead serve as examples of different ways in which agentic capabilities are being introduced into bioinformatics. Particular attention was given to how the literature describes analytical autonomy, reproducibility, validation, provenance, biological interpretation, and human oversight.

Direct performance comparisons were considered inappropriate because the reported systems differ substantially in their biological objectives, architectures, data modalities, analytical tasks, and intended uses. A system developed for single-cell annotation, for example, cannot be meaningfully judged using the same performance criteria as one designed for microbial interpretation, biomarker discovery, or general multi-omics workflow execution. The review therefore focuses on recurring scientific and reporting issues rather than attempting to establish an overall ranking of existing platforms.

This descriptive synthesis also informed the broader conceptual questions developed in the manuscript: which analytical decisions may be delegated to an agent, when those decisions become scientifically consequential, what forms of validation are appropriate for the resulting claims, how supporting evidence can be preserved, and where human review remains necessary. The descriptive characteristics extracted for the agentic bioinformatics systems discussed in this review, including biological application, data modality, agent architecture, analytical role, validation approach, human oversight, provenance features, and code or data availability, are summarized in Supplementary Table S1.

### 2.3. Evidence Base and Scope

The evidence base was restricted to peer-reviewed, PubMed-indexed publications relevant to the scope of the review. Within this literature, original peer-reviewed publications describing implemented agentic bioinformatics systems that met the criteria above were selected for more detailed discussion. Information that could not be verified from the available sources was treated conservatively as not reported.

The broader literature was used to examine established issues in bioinformatics reproducibility, statistical inference, provenance, biological validation, governance, and reporting. The selected agentic systems were used primarily as illustrative examples of how analytical autonomy, validation, provenance, and human oversight are currently described across different bioinformatics applications. Accordingly, the review does not attempt to provide a formal ranking or quantitative comparison of these systems.

Instead, the published evidence is used to identify recurring practices, limitations, and reporting gaps that motivate the concepts developed in the subsequent sections, including delegated decision rights, consequence-sensitive validation, claim-to-evidence traceability, and the proposed THREAD-Bio framework.

## 3. Biological Applications and Evidence Practices of Agentic Bioinformatics

Agentic bioinformatics systems differ substantially in their biological objectives, analytical authority, and evidential requirements. Some systems recommend workflows or explain genomic concepts, whereas others select tools, execute analyses, revise plans, and interpret results. Their outputs may concern cell identity, gene regulation, antimicrobial resistance, viral risk, biomarker discovery, predictive modelling, or clinical interpretation. Evaluating these systems solely according to task completion therefore overlooks the scientific consequences of the decisions they influence.

Published systems already incorporate safeguards such as restricted execution environments, interaction logs, source identifiers, automated validation, critic agents, quality scores, and expert assessment. However, these safeguards address different parts of the analytical process. An execution check does not establish that the study design was appropriate. A literature citation does not necessarily support every biological statement derived from it. Agreement among multiple agents does not replace independent biological validation. This section examines the biological questions addressed by current systems, the evidence they preserve, and the remaining gap between computational output and biological justification.

### 3.1. Biological Questions Addressed by Current Systems

General-purpose bioinformatics agents operate across several analytical domains. AutoBA [15] constructs analytical plans, generates code, executes workflows, and repairs failed operations across multiple omics tasks. Its decisions may affect which samples pass quality control, which features are retained, and which pathways are reported. BRAD [16] combines literature retrieval, biological databases, and analytical modules for biomarker discovery. Its outputs therefore depend on the phenotype definition, selected analytical strategy, and relevance of the retrieved evidence. BioAgents [17] provides conceptual genomics support and workflow recommendations. BioMedAgent [18] coordinates tools across cross-omics, machine-learning, and biomedical-imaging analyses. These systems differ in their scientific authority because a recommendation can be reviewed before execution, whereas an executing agent may propagate an unsuitable reference, sample grouping, or analytical method through several downstream stages.

Single-cell systems address questions involving cellular identity, heterogeneity, and disease-associated states. CellVoyager [1] explores single-cell RNA-sequencing data, develops hypotheses, and implements analyses in computational notebooks. Its outputs may influence whether a cluster is interpreted as a known lineage, a transient activation state, a doublet, or a rare disease-associated population. CASSIA [21] distributes cell-type annotation across annotator, validator, scorer, and reporter agents. A plausible annotation nevertheless requires coherent marker expression and exclusion of incompatible lineages or technical artifacts. Agentomics [19] explores predictive models and hyperparameters under programmatic checkpoints. BIOGEN [22] retrieves and evaluates evidence when interpreting bacterial transcriptomic clusters associated with antimicrobial resistance. These systems evaluate different biological objects, including cellular identity, predictive phenotype, and gene-program meaning. Their validation requirements should therefore reflect the specific claim being produced rather than a generic measure of agent performance.

Microbial and cancer applications carry additional evidential consequences. Viral Sentry AI [23] integrates viral-genome surveillance, host-risk prediction, and downstream drug-repurposing analysis. A predicted risk threshold may therefore initiate another analytical process with potential therapeutic implications. AI-HOPE-WNT [24] supports conversational analysis of WNT-pathway dysregulation in colorectal cancer. AI-HOPE-PM [25] integrates genomic, clinical, and social variables in precision-medicine analyses. Lense [29] supports single-cell preprocessing and exploration. OMICX [30] supports multi-condition transcriptomic analysis. Related systems extend to genomic visualization, protein analysis, functional annotation, spatial omics, and plant biology. The distinction between an AI-assisted analytical platform and an agentic system therefore depends on what the system can do rather than on the label applied to it. In this review, systems were considered agentic when they could determine or revise part of the analytical route through planning, tool use, iterative evaluation, or coordinated action, as described in Section 2. Systems are therefore discussed according to the biological decisions they can influence rather than solely according to their architecture or self-applied label.

### 3.2. Reported Validation, Evidence, and Safeguards

Several systems include safeguards directed primarily at computational integrity. AutoBA [15] operates within a sandbox, restricts executable commands, reports failed cases, and includes bioinformatician review of generated plans, code, and outputs. These measures make automated repair more visible and reduce the risk of unrestricted execution. BRAD [16] preserves detailed interaction and decision logs while linking biomarker-related responses to cited literature. Agentomics [19] uses a restricted container, hidden test labels, deterministic performance metrics, and artifact validation. These controls reduce the likelihood that leaked labels, invalid files, or undocumented changes will produce misleading performance claims.

Interpretive systems preserve different forms of biological evidence. BIOGEN [22] retains source identifiers, intermediate evidence, critic assessments, evidence tiers, and stated limitations for bacterial transcriptomic interpretation. The presence of a source identifier demonstrates that the source can be located, but it does not establish that every sentence is directly supported or biologically correct. CASSIA [21] records conversations, validation decisions, supporting evidence, quality scores, and low-quality annotations. It can revise annotations that fail internal checking and flag uncertain clusters for further review. CellVoyager [1] incorporates expert assessment of case-study outputs. BioMedAgent [18] reports reference steps and analytical milestones across benchmark tasks. These mechanisms address different questions, including whether evidence exists, whether an annotation is internally coherent, whether an expert considers an interpretation plausible, and whether a workflow reproduces an expected output.

Execution checks, predictive metrics, expert assessment, and biological validation should not be combined under a single label of validation. Execution checks establish whether commands and interfaces operate correctly. Deterministic metrics assess predictive or computational performance under specified conditions. Expert assessment evaluates whether an output appears plausible. Independent biological assays or external cohorts determine whether a proposed phenotype, mechanism, or clinical interpretation is supported beyond the originating analysis. A system may perform strongly at one level while remaining inadequately validated at another.

These approaches also address different aspects of reliability and reproducibility. Restricted execution environments and artifact checks primarily support computational reliability, whereas interaction logs, recorded analytical decisions, and preserved source identifiers can improve the ability to reconstruct an analysis. Expert assessment provides an additional check on biological plausibility but is not equivalent to independent biological or external validation. The reviewed reports varied in the extent to which validation beyond the original development or case-study setting was described, making direct comparison of external validation inappropriate. Similarly, adoption beyond the settings described in the original publications was not consistently reported and was therefore not compared quantitatively in this review.

### 3.3. Remaining Gaps in Biological Justification and Accountability

Published reports generally describe computational execution more clearly than delegated scientific authority. Autonomous execution, interpretation, and reporting were commonly described among the systems discussed in this review, whereas authority over data selection, parameter choices, and statistical decisions was less consistently reported. Across the examined publications, delegated authority was generally not presented in a single comparable form covering data, methods, parameters, statistics, execution, interpretation, and reporting together with permission boundaries, approval conditions, reversibility, scientific consequences, and data sensitivity. This does not demonstrate that permissions were uncontrolled in practice. It indicates that published reports did not present them in a comparable form.

Scientific abstention and human oversight were also incompletely described. Several systems reported partial mechanisms for handling unsuccessful or uncertain analyses, including failure labels, retry ceilings, trigger thresholds, low-quality flags, or evidence tiers. However, explicit scientific abstention rules that would prevent a technically runnable but biologically unsupported analysis from being presented as a strong biological claim were rarely described. AutoBA [15] includes human intervention and expert review. Several other systems involve specialists during development or evaluation. However, post hoc expert assessment is not equivalent to a human approval gate during the analytical process. A human approval gate should identify the consequential decision, the evidence required for approval, the responsible reviewer, and the conditions under which the analysis may continue.

Across the published reports examined here, a complete claim-to-evidence connection was not consistently described from each biological claim back to its underlying data, analytical decisions, computational actions, and supporting evidence. BRAD [16] approaches this requirement through detailed decision logs and literature citations. BIOGEN [22] approaches it through source identifiers, evidence tiers, and critic records. CASSIA [21] approaches it through annotation histories, validation decisions, and quality scores. These systems provide important foundations rather than examples of complete failure. The unresolved problem is connectivity. A reader should be able to move from a statement about a biomarker, cell identity, resistance mechanism, or viral risk to the interpretation, statistical result, analytical decision, software execution, reference resource, input samples, and original biological question that produced it. Table 1 summarizes the biological applications and the validation, evidence, and oversight features described in the published reports.

## 4. How Agentic Failures Distort Biological Conclusions

Failures in agentic bioinformatics do not arise from a single source. Some originate from familiar weaknesses in data quality, study design, statistical analysis, or biological interpretation. Others occur when autonomous execution propagates an early mistake across multiple analytical stages. A third category emerges from agent-specific mechanisms, including memory, retrieval, tool interaction, and coordination among multiple agents. Distinguishing these origins is important because each requires different forms of prevention, detection, validation, and oversight.

These categories are not mutually exclusive. A conventional bioinformatics error may be amplified through repeated autonomous actions and further obscured by automated interpretation or reporting. For example, an incompatible reference genome may first affect alignment, then propagate into annotation and downstream analysis, and finally appear in a polished biological narrative. The resulting workflow may be technically complete while supporting a scientifically misleading conclusion.

The available literature does not yet provide sufficiently standardized reporting to estimate how frequently specific failures occur across agentic bioinformatics systems. Published studies differ substantially in their tasks, datasets, validation procedures, and definitions of error, and many report performance rather than the frequency of biologically consequential failures. Quantitative failure rates should therefore not be generalized across systems without task-specific benchmarking. The more immediate requirement is to define how each type of failure can be detected and what scientific consequences may follow when it is not identified.

### 4.1. Inherited Biological and Bioinformatics Failures

Inherited failures are problems that can occur without the involvement of an artificial intelligence agent. Input and reference errors include corrupted files, sample swaps, contamination, incomplete metadata, incompatible genome builds, and incorrect organism or annotation resources. Workflow errors include unsuitable normalization, outdated software, inconsistent file formats, and undocumented defaults. Statistical failures include confounding, data leakage, unmodelled dependence, invalid contrasts, uncontrolled multiple testing, and post hoc selection. Interpretation failures include causal claims derived from association, enrichment analyses using inappropriate background sets, and mechanistic conclusions supported only by biological plausibility. These risks are well documented in sequencing laboratories, multi-omics integration, microbiome research, and cancer biomarker analysis [3,6,8,31].

Single-cell analysis illustrates how inherited errors can produce convincing but invalid findings. Cell filtering may remove biologically relevant populations or retain low-quality droplets. Doublets may appear as artificial intermediate states, whereas integration may remove genuine biological differences. Annotation may become circular when the same markers or references are used to assign and validate cell identities. Differential-expression analysis may also treat individual cells as independent replicates, creating pseudoreplication and exaggerated statistical precision. Established frameworks address integration, doublet detection, annotation, and differential analysis in multi-sample single-cell studies [9,32,33,34,35]. However, these methods do not remove the requirement that independent evidence must be defined by donors or experimental units rather than by the number of cells.

Detection therefore depends on checks appropriate to the biological task. These may include confirming sample and reference compatibility, inspecting quality-control metrics, preserving biological replication in statistical models, checking cell annotations against established marker evidence, and comparing computational interpretations with independent databases or experimental knowledge. Failure to detect these problems may lead to incorrect cell identities, spurious differential signals, misleading pathway associations, or biological conclusions that are stronger than the underlying evidence supports.

### 4.2. Amplification of Error During Autonomous Analysis

Agentic systems can increase the speed, scale, and invisibility of inherited errors. An incorrect genome build selected at the beginning of an analysis may be propagated through alignment, annotation, visualization, and manuscript drafting without independent review. An unsuitable default may be applied across several cohorts, whereas an invalid contrast may be repeated at multiple analytical resolutions. Portable and scalable workflow systems improve reproducibility and computational efficiency across environments [36,37,38]. However, when planning and execution are delegated to an agent, the same scalability can multiply the consequences of an inappropriate analytical choice.

Automated retry creates a particularly important amplification pathway. Correcting a syntax error, missing dependency, or malformed path is primarily a technical repair. In contrast, changing a filtering threshold, reference genome, annotation database, clustering resolution, or statistical model may alter the biological meaning of the result. If only the final successful attempt is retained, the reported finding may conceal the analytical alternatives explored during execution. Repeated adjustment against intermediate outputs can also create analytical overfitting when an agent continues until a cluster appears coherent, a comparison becomes statistically significant, or a pathway becomes biologically interpretable. Agentomics [19] limits part of this risk through hidden test labels and deterministic evaluation metrics. AutoBA [15] exposes failed cases and automated repair cycles. Nevertheless, retries that affect biological interpretation should be recorded as scientific decisions rather than treated solely as software maintenance.

Automated interpretation and reporting may further amplify weak inference. A polished figure, pathway narrative, or generated conclusion can appear more authoritative than the underlying evidence warrants. Retrieval-augmented generation may improve attribution, but retrieved material can still be outdated, irrelevant, or inconsistent with the specific claim being made [39]. Foundation-model applications in single-cell and protein analysis similarly require task-specific validation rather than reliance on fluent or coherent explanations [40,41]. Amplification therefore arises from coupling: the same system may plan, execute, select, interpret, and report an analysis without sufficient independent scrutiny between these stages.

Amplification can be detected by preserving intermediate results, recording retries and parameter changes, comparing revised analyses with the original analytical plan, and repeating consequential analyses under fixed or independently reviewed settings. The scientific impact can exceed that of the initial error because one inappropriate choice may propagate across several downstream outputs and ultimately influence multiple figures, interpretations, or reported claims.

### 4.3. Emergent Failures from Memory, Retrieval, Tool Use, and Coordination

Emergent failures arise specifically from agentic mechanisms. Objective drift occurs when iterative planning gradually replaces the original biological question with an easier computational task. Context loss occurs when paired designs, exclusion criteria, sample constraints, negative controls, or other essential study features disappear from the active working context. Stale memory may reintroduce outdated references or assumptions from earlier tasks. Retrieval may expose the system to misleading documentation, obsolete database records, or instructions embedded within software repositories and metadata. Tool descriptions may also be incomplete, leading an agent to assume that an unavailable capability or interface exists.

Tool use introduces additional security and provenance risks. An agent may be authorized to read sensitive files, install packages, overwrite intermediate outputs, or access external networks. Local deployment may reduce data transfer to external model providers, but it does not automatically ensure least-privilege access, immutable activity records, or secure software dependencies. Conversely, restricting network access may prevent retrieval of current evidence and result in outdated interpretation. Autonomy, external tools, and persistent memory create recognized security risks in large-model agents [28]. In bioinformatics, these concerns are intensified by identifiable genomic data, clinical phenotypes, mutable biological databases, and long software supply chains. Sandboxing, command allowlists, read-only storage, and isolated credentials should therefore be reported as specific permission controls rather than summarized using a general claim that a system is secure.

Multi-agent systems introduce coordination failures that cannot be resolved merely by counting votes. Planner, executor, and evaluator agents may share the same model family, training data, prompt assumptions, or retrieval sources. Agreement among them may therefore reproduce the same incorrect marker, pathway interpretation, or gene–phenotype assumption. Information passed between agents may also lose caveats concerning donor structure, organism context, uncertainty, or contradictory controls. CASSIA [21] uses specialized roles to expose intermediate cell-annotation checks. BIOGEN [22] uses multiple critics to evaluate bacterial transcriptomic interpretations. Their principal value lies in making intermediate evaluation more visible; agreement among agents does not independently establish a cell identity or antimicrobial-resistance mechanism.

**Figure 2:**
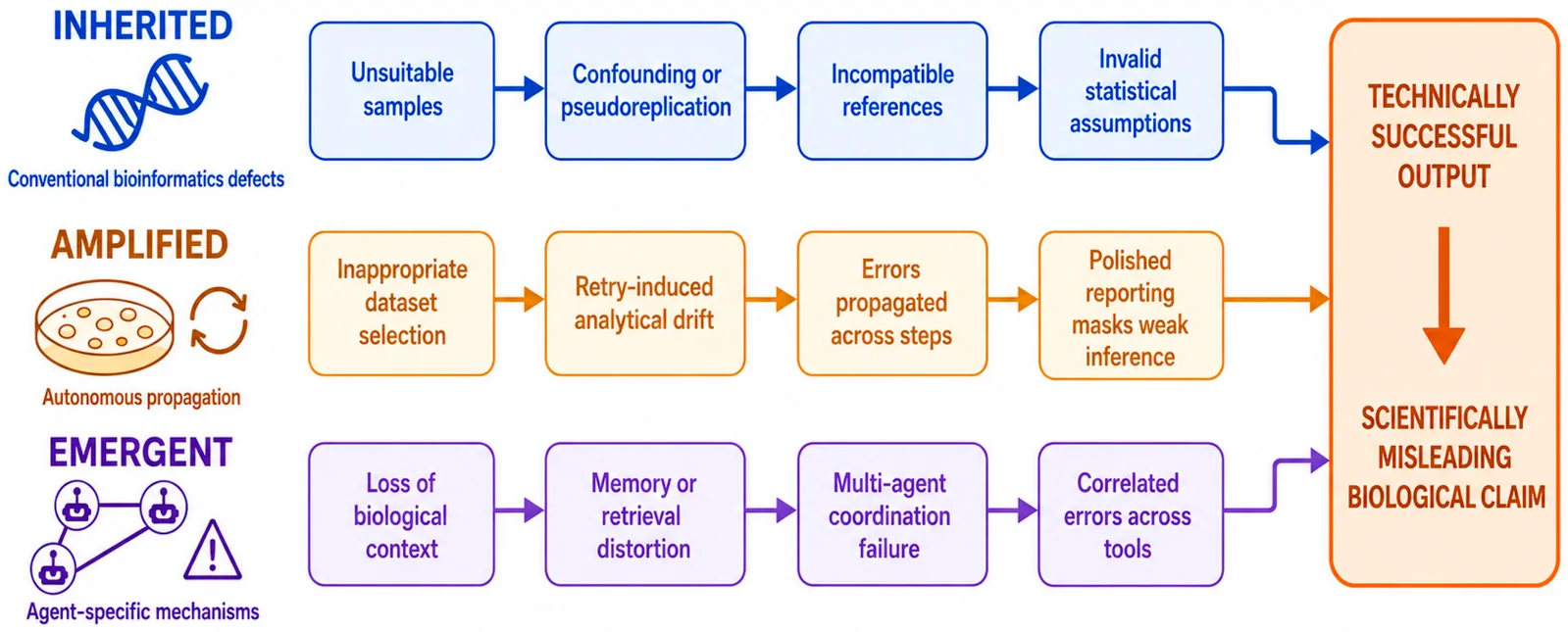
Inherited, amplified, and emergent failure pathways in agentic bioinformatics. Conventional bioinformatics errors may enter an agent-assisted workflow through unsuitable data, references, methods, statistical models, or interpretations. Autonomous execution can amplify these errors through repeated actions, parameter changes, propagation across analytical steps, and automated reporting. Agent-specific failures may also arise from objective drift, loss of biological context, outdated or misleading retrieval, unsafe tool use, and correlated errors among multiple agents. These pathways can converge on technically successful outputs that nevertheless support scientifically misleading biological conclusions.

Detection of emergent failures requires inspection of the agent’s recorded actions and evidence rather than assessment of the final output alone. The original research objective can be compared with later analytical decisions to identify objective drift; retrieved evidence can be checked against its source and version; tool calls and file changes can be reviewed through execution records; and multi-agent conclusions can be compared with independent biological evidence rather than accepted on the basis of agreement alone. If these failures remain undetected, they may produce outdated, poorly supported, or internally consistent but biologically incorrect interpretations. Figure 2 summarizes how inherited, amplified, and emergent failures can converge on technically successful outputs that nevertheless support scientifically misleading biological conclusions.

## 5. Delegated Decisions and Human Oversight in Biological Analysis

Agentic bioinformatics systems cannot be adequately described using a single autonomy score. Two systems may both be labelled autonomous while exercising substantially different forms of scientific authority. One system may execute a researcher-approved workflow without changing its design, whereas another may select datasets, modify parameters, revise statistical models, interpret results, and draft biological conclusions. These differences determine whether an automated action merely affects computational execution or changes the scientific meaning of the analysis.

Delegated authority should therefore be described at the level of individual decisions. The proposed conceptual decision-rights profile separates authority across data selection, methods, parameters, statistics, execution, interpretation, and reporting. Each action is further considered according to its permission scope, reversibility, scientific consequence, and data sensitivity. This approach distinguishes what an agent is technically capable of performing from what it is scientifically authorized to decide. It also identifies the transitions at which a human approval gate or independent review should be required.

### 5.1. Decision Rights Across the Biological Workflow

The data domain includes dataset discovery, sample eligibility, exclusion criteria, and construction of comparison groups. These decisions determine which donors, tissues, organisms, or disease states are represented in the analysis. The methods domain includes workflow selection, software, reference genomes, annotation resources, and analytical strategies. The parameters domain covers quality-control thresholds, clustering resolutions, variant filters, and database versions. Decisions in these domains can directly alter which samples or biological features are retained, excluded, or interpreted.

The statistics domain includes the estimand, contrast, covariates, dependence structure, multiplicity correction, missing-data handling, and unit of replication. The execution domain covers commands, automated retries, file access, package installation, network use, and modification of intermediate outputs. Interpretation includes the assignment of cell identities, pathways, mechanisms, resistance phenotypes, causal meaning, or clinical relevance. Reporting determines which findings are included in figures, summaries, conclusions, and manuscript text. Human involvement in transcriptomic analysis may range from methodological guidance to biological interpretation and final decision-making [42]. Interactive computational systems may also incorporate expert input during exploratory analysis and data selection [43].

Authority within each domain can be described as absent, recommendation only, action requiring human approval, autonomous action within a defined policy, unbounded, unclear, or not reported. This vocabulary separates capability from permission. For example, an agent may be technically capable of changing a clustering resolution or statistical contrast, but such a change may still require human approval because it affects the interpretation of a cell population or disease association. Table 2 summarizes the seven decision domains, representative risks, and evidence required to reconstruct delegated authority.

### 5.2. Reversibility, Scientific Consequence, and Data Sensitivity

Decision type alone does not determine risk. Reversibility describes whether an action can be undone without losing provenance or invalidating downstream work. Changing a plot label is readily reversible, whereas overwriting a unique intermediate file or disclosing protected genomic data may not be. Scientific consequence describes how an error would affect the study. Selecting a visualization format is generally less consequential than redefining the cohort, changing the primary contrast, or introducing causal language into a conclusion. Data sensitivity describes whether the action involves public data, restricted research data, clinical information, or identifiable genomic material.

These modifiers should be considered together. An agent may safely explore alternative clustering resolutions when every result is retained, clearly labelled as exploratory, and generated from a copied data object. The same action becomes higher risk when the selected resolution determines a clinically relevant cell-state claim and the discarded alternatives are not preserved. Network access may be appropriate for retrieving public software documentation but unacceptable when the working environment contains protected patient data. Retrieval-supported interpretation in oncology can provide access to current evidence, but it also creates concerns involving privacy, attribution, relevance, and evidential quality [39].

The proposed profile therefore assigns risk to individual actions within their specific scientific and data contexts. It avoids describing an entire system as simply high autonomy, low autonomy, secure, or unsafe. A low-consequence action may proceed autonomously under a defined policy, whereas a scientifically consequential or irreversible decision should require stronger validation and a human approval gate. This action-specific approach allows automation to be retained without granting unrestricted authority across the entire analytical workflow.

### 5.3. Human Approval Gates for Consequential Decisions

Effective human oversight requires more than placing a researcher somewhere within the workflow. A human approval gate should identify the decision being reviewed, the responsible reviewer, the evidence presented, the conditions for approval or rejection, and any subsequent override. Mandatory approval is most appropriate when an action changes the study population, primary estimand, genome build, inferential model, causal interpretation, clinical meaning, or public release of sensitive information. Routine execution may proceed under a predefined policy when inputs, permissions, and integrity checks satisfy established conditions.

Human review should not be treated as infallible. Experts may share methodological biases, overlook implementation errors, or approve familiar but unsuitable approaches. The purpose of a human approval gate is not to position human judgement as inherently superior to computation. Its purpose is to create an accountable transition at which assumptions, evidence, uncertainty, and possible consequences can be inspected before the analysis proceeds. Generative artificial intelligence should support rather than replace responsible statistical judgement [44]. Human participation in scientific publishing should also remain connected to methodological responsibility rather than ceremonial approval or authorship [45].

Oversight should itself be evaluated. Relevant measures include reviewer disagreement, time burden, override frequency, the number and type of errors detected, and whether approval improves the calibration of final claims. A human approval gate that adds substantial administrative burden without identifying consequential problems may require simplification. Conversely, a gate that repeatedly detects invalid cohort definitions, unsupported statistical contrasts, or overstated clinical interpretations provides evidence of practical value. Consequence-sensitive oversight therefore treats human review as a testable component of the analytical system rather than an assumed guarantee of trustworthiness.

## 6. Tracing Biological Claims Back to Data and Evidence

Reproducibility in bioinformatics traditionally concerns whether an analysis can be repeated using the same data, software, parameters, and computational environment. This information is essential for verifying how a count matrix, variant set, phylogenetic tree, or other analytical output was generated. However, reproducing a computational result does not necessarily establish that the result supports the biological interpretation assigned to it.

This distinction becomes more important in agentic bioinformatics because an agent may revise its analytical plan after inspecting intermediate outputs. A complete record must therefore preserve not only computational operations but also the decisions, evidence, validation outcomes, interpretations, and approvals that connect the original biological question to the final claim. This section distinguishes conventional workflow provenance from biological justification and proposes a claim-to-evidence structure that links both.

### 6.1. Limits of Conventional Workflow Provenance

Bioinformatics provenance can record input files, software versions, parameters, computing environments, intermediate artifacts, and final outputs. The FAIR principles provide a foundation for making research objects findable, accessible, interoperable, and reusable [46]. CWLProv connects computational entities, activities, and responsible agents within structured workflow-provenance records [47]. BioCompute Objects provide standardized descriptions of computational analyses, including their inputs, execution domains, error handling, and outputs [48]. Provenance capture has also been implemented in genomic workflows, pipeline documentation systems, and provenance-aware data-acquisition platforms [49,50,51].

Computational lineage nevertheless answers a different question from biological justification. A workflow record may explain how a microbial abundance table was produced without demonstrating why a detected taxon was interpreted as functionally important. It may reconstruct a single-cell embedding without establishing that a cluster represents a distinct biological state. It may regenerate a phylogenetic tree without proving direct transmission between patients or hospital locations. QIIME 2 Provenance Replay [52] can reconstruct microbiome-analysis steps. Containerized environments preserve software dependencies and computational configurations [53]. Project-based frameworks organize code, data, and outputs within reproducible analytical structures [54]. These resources strengthen computational reproducibility, but they do not automatically provide the evidential argument connecting an output to a biological claim.

Provenance requirements also vary across biological domains. Reproducible workflows have been developed for bacterial genome assembly [55], proteomics [56], spatial transcriptomics [57], genome-wide association analysis [58], and public-health genomics [59]. Sustainable FAIR implementation additionally depends on software maintenance, community standards, and adequate documentation [60,61,62]. These approaches provide the computational foundation for agentic traceability. The remaining requirement is to represent changing objectives, delegated decisions, retrieved evidence, and scientific claims as explicit provenance objects.

### 6.2. The Claim-to-Evidence Graph

The proposed claim-to-evidence graph provides a conceptual structure for tracing a specific biological claim upstream through its interpretation, statistical result, analytical decision, computational execution, reference resource, input samples, and original research question. A statement that a cell population is exhausted, a bacterial isolate is resistant, or a tumour variant is clinically actionable must remain linked to the evidence required for that particular assertion. Each node should contain a stable identifier, timestamp, version, responsible human or agent, evidence state, and human-approval state where applicable. Under W3C PROV terminology, data, software, results, and claims may be represented as entities; retrieval, analysis, and interpretation may be represented as activities; and researchers, institutions, models, and software services may be represented as agents.

Claims should be separated when they require different evidential thresholds. A single paragraph may contain an observation, an association, a proposed mechanism, and a clinical implication. These statements should not inherit the same evidence status merely because they appear together. BIOGEN [22] links bacterial transcriptomic interpretations to source identifiers and evidence records while distinguishing source traceability from biological correctness. CASSIA [21] preserves annotation conversations, validation decisions, supporting evidence, and quality scores. BRAD [16] retains operational decisions and literature sources during biomarker analysis. These systems provide partial implementations of claim-level traceability. The proposed graph extends these practices by requiring each biological assertion to remain connected to the data, analytical decisions, results, and evidence from which it was derived.

**Figure 3:**
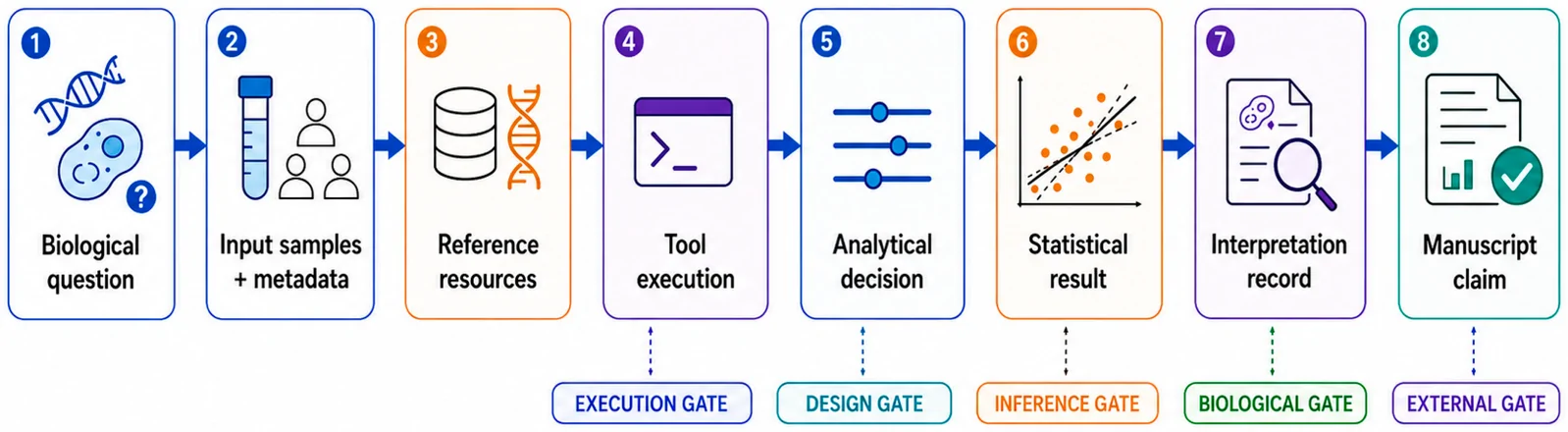
Claim-to-evidence traceability architecture for agentic bioinformatics. The framework links the original biological question to input samples and metadata, reference resources, computational actions, analytical decisions, statistical results, interpretation records, and the final scientific claim. Execution, design, inference, biological, and external validation gates identify the stages at which evidence should be evaluated before a claim progresses. Provenance records preserve the versions, responsible agents or researchers, evidence states, and approval states associated with consequential transitions. THREAD-Bio integrates these elements into a connected reporting structure that allows the route from data to claim to be reconstructed, evaluated, and challenged.

For example, a claim that a bacterial lineage transmitted between two hospital wards should link to the phylogenetic or genomic-distance result, the relatedness threshold, the selected isolates, sampling dates, assembly and variant-calling workflow, reference genome, and epidemiological question. A reproducible tree alone cannot reveal whether the transmission claim exceeds the resolution of the available data. Similarly, a claim that a single-cell cluster represents an activated T-cell state should link to marker statistics, contradictory markers, annotation evidence, clustering choices, integration decisions, donor identities, and the original comparison. Figure 3 illustrates this architecture by connecting the biological question, data, reference resources, computational actions, analytical decisions, statistical results, interpretation records, validation gates, and final manuscript claim.

### 6.3. Decision Records, Evidence States, and Human-Intervention Records

Decision provenance does not require disclosure of private model reasoning or unrestricted internal chain-of-thought. A concise decision record can identify the decision, alternatives considered, rule or evidence applied, uncertainty, selected action, approver, and affected downstream artifacts. For example, a record may state that GRCh38 was selected because the sample coordinates and annotation resource used that genome build, whereas GRCh37 was rejected after compatibility checking. The same format can document a changed clustering resolution, substituted software tool, excluded sample, revised statistical contrast, or modified biological interpretation.

Evidence states prevent different forms of support from being treated as equivalent. A result may be technically verified, statistically supported, biologically plausible, contradicted, externally replicated, or experimentally validated. Retrieved literature may provide direct support, indirect support, conflicting evidence, or outdated information. A critic score should therefore indicate what was evaluated rather than being interpreted as the probability that a biological mechanism is true. Human-intervention records should likewise distinguish a user instruction, optional feedback, expert assessment, mandatory human approval, rejection, and override. Structured documentation is necessary for preserving analytical context, supporting reviewability, and maintaining sustainable research software [50,62].

The resulting graph can be serialized in a machine-readable format and linked to existing workflow objects. Data manifests can retain sample identifiers, eligibility decisions, exclusion records, and metadata versions. Reference manifests can record genome builds, annotation releases, database versions, and retrieval dates. Statistical objects can preserve model specifications, contrasts, diagnostics, multiplicity procedures, and uncertainty. Claim objects can link directly to manuscript sentences, figures, or table entries. Galaxy [63] demonstrates how structured workflow artifacts can support reproducible analysis. Standardized metagenomic workflows can similarly preserve computational records across analytical stages [64]. Public-health genomics platforms show how structured analytical artifacts can be integrated into operational surveillance systems [59]. The agent-specific addition is a connected layer of decisions, evidence, validation states, approvals, and claims rather than a replacement of established provenance infrastructure.

## 7. Validation Gates and Calibrated Abstention

Validation in agentic bioinformatics should not be treated as a single pass-or-fail judgement. A computational operation may execute successfully while the underlying study design is unsuitable, the statistical inference is invalid, or the biological interpretation lacks independent support. Different forms of validation therefore address different questions and should be reported separately.

The proposed framework distinguishes five validation gates: execution, design, inference, biological, and external validation. Each gate specifies the evidence required for an analysis or claim to proceed. The threshold for passing a gate should be defined according to the biological task, study design, intended use, and consequence of the resulting claim rather than by a universal numerical cut-off. Where quantitative criteria are appropriate, such as quality-control limits, model diagnostics, predictive-performance requirements, or replication criteria, they should be specified before the relevant result is interpreted. For qualitative decisions, the required evidence and conditions for human approval should likewise be stated in advance.

Failure at a gate does not necessarily require the system to terminate all computation. Instead, the agent may continue with diagnostic, descriptive, or exploratory analyses while restricting the evidential status of the resulting output. This calibrated form of abstention prevents technically valid outputs from being presented as stronger biological conclusions than the available evidence supports.

### 7.1. Execution and Design Gates

The execution gate determines whether a computational operation completed correctly. Relevant evidence includes command status, expected output files, schema validation, non-empty results, plausible value ranges, software versions, and documented retry history. A command that exits without an error may still produce an unusable output, such as a truncated variant file, an empty marker table, incompatible identifiers, or corrupted intermediate data. These failures should prevent downstream interpretation until the integrity problem has been resolved.

Agentomics [19] uses automated interface checks, deterministic metrics, hidden test labels, and artifact validation to assess computational integrity. AutoBA [15] operates within a restricted environment and records failed operations and repair attempts. These safeguards establish that tools and interfaces function as intended. However, they do not determine whether the selected samples, biological comparison, or analytical objective are scientifically appropriate.

The design gate evaluates whether the proposed analysis can answer the biological question. It examines cohort composition, experimental units, paired or longitudinal structure, comparison groups, covariates, batch effects, metadata completeness, reference compatibility, and whether the intended estimand can be identified from the available data. Missing group labels, perfect confounding, incompatible genome builds, or absent biological replication should trigger abstention rather than automated improvisation. Legacy and heterogeneous datasets may introduce confounding even when their files are technically valid [65]. Clinical genomic interpretation likewise depends on explicit context, sample characteristics, and reporting standards [66].

For both gates, passing should mean that the predefined requirements relevant to the intended analysis have been satisfied, not simply that no software error was detected. A failed execution gate should prevent interpretation of the affected output, whereas a failed design gate should prevent claims that depend on a comparison the available data cannot support.

### 7.2. Inference Gate

The inference gate evaluates whether statistical assumptions, uncertainty estimates, and inferential claims are defensible. The record should identify the model, estimand, contrast, covariates, dependence structure, multiplicity procedure, missing-data strategy, diagnostics, and sensitivity analyses. A paired study analysed as independent, individual cells treated as biological replicates, leakage between training and testing data, or a post hoc comparison presented as confirmatory should fail this gate. Appropriate statistical inference in multi-sample single-cell studies depends on retaining donor-level replication and modelling dependence between cells from the same biological sample [33,67].

Statistical validity must be evaluated separately from code correctness. A model may be implemented correctly but remain inappropriate for the study design. A stable result may reflect confounding rather than a robust biological association. Sensitivity analysis is informative only when it examines scientifically consequential choices, such as alternative covariates, thresholds, reference resources, or model structures. Benchmarking studies have shown that the performance of normalization, imputation, isoform quantification, and single-cell proteomic methods depends on the characteristics of the data and evaluation framework [68,69,70,71]. Multi-omics integration also requires explicit consideration of scale, missingness, and cross-modal alignment rather than assuming that a joint representation resolves these issues [3].

Failure at the inference gate should produce calibrated abstention. An agent may report that the analysis is underidentified, that the metadata are insufficient, or that the result should be considered exploratory. It may still provide diagnostic plots, descriptive summaries, or hypothesis-generating outputs. A pathway list may therefore be reported as exploratory without supporting a mechanistic conclusion. A classifier may be described as internally validated without being presented as clinically useful. Abstention limits the claim rather than requiring complete silence.

The relevant threshold should therefore reflect the type of inference being made. Exploratory analyses may proceed with clearly stated uncertainty, whereas confirmatory, causal, translational, or clinical claims require progressively stronger statistical support and independent evidence.

### 7.3. Biological and External Gates

The biological gate evaluates whether the interpretation is supported by appropriate controls, established biological knowledge, contradictory evidence, and independent sources. In single-cell annotation, proposed identities should be assessed using coherent marker expression, incompatible lineage markers, doublet signatures, and relevant reference atlases [21,72,73]. In biomarker studies, agreement across transcriptomic, proteomic, or other molecular layers may strengthen an interpretation, but convergence does not replace independent replication or functional evidence [74,75,76,77]. Active searches for contradictory evidence are also necessary because retrieval systems may otherwise preferentially collect sources that support the selected explanation.

The external gate asks whether support extends beyond the originating analysis. Depending on the claim, external evidence may include an independent cohort, another analytical platform, an orthogonal assay, a perturbation experiment, functional validation, or prospective clinical replication. Evaluation on an additional benchmark may support general task performance without validating a specific biological mechanism. CASSIA [21] evaluates cell-type annotation across multiple datasets, strengthening evidence for annotation performance. BIOGEN [22] evaluates traceable bacterial transcriptomic interpretation under distribution shift. However, these evaluations do not automatically establish that every proposed cell identity or antimicrobial-resistance mechanism has been experimentally validated.

The threshold for biological or external validation should therefore be proportional to the claim. A descriptive observation may require internal consistency and appropriate controls, whereas a mechanistic claim requires stronger biological support, and a translational or clinical claim should not proceed without evidence appropriate to its intended use.

Human approval requirements should increase as claims progress through the five gates. Routine execution may be cleared through automated integrity checks. Cohort definitions, exclusion criteria, and principal contrasts should be reviewed by an appropriate domain expert. Consequential statistical claims may require statistical review, while mechanistic, causal, translational, or clinical conclusions require explicit assessment of biological and external evidence. Strong clinical claims should be withheld when proportional external support is absent. Table 3 summarizes the central question, minimum evidence, failure condition, and human approval requirement associated with each validation gate.

### 7.4. Implementing Calibrated Abstention and Continuous Validation

In practice, calibrated abstention can be implemented by linking each validation gate to a limited set of permitted outcomes. An analysis may pass and proceed, require revision or additional evidence, or continue only with a restricted claim. For example, failure of external validation need not prevent an agent from reporting an internally supported association, but it should prevent the same result from being presented as a validated biomarker or clinical recommendation. Similarly, failure of an inference gate may still permit descriptive summaries while withholding confirmatory or causal language.

The reason for abstention should also be retained as part of the analytical record. This should identify the gate that was not satisfied, the evidence that was missing or contradictory, the outputs that remain permissible, and whether human review is required before the analysis proceeds. In this way, abstention becomes an explicit limitation on scientific interpretation rather than a generic refusal to produce an answer.

Maintaining validation throughout an iterative agentic workflow nevertheless introduces practical challenges. Repeated checks increase computational cost and may slow analyses, particularly when agents revise parameters, rerun workflows, retrieve updated resources, or explore multiple analytical alternatives. Reference databases and software versions may change during long-running analyses, while validation criteria appropriate to one data type or biological question may not transfer directly to another. Excessive validation can also create unnecessary human-review burden if every minor computational action requires approval.

A practical approach is therefore to repeat validation when a scientifically consequential change occurs rather than after every computational step. Changes to the dataset, reference resource, primary method, statistical model, principal contrast, biological interpretation, or intended strength of the claim should trigger re-evaluation of the relevant gate. Routine technical operations can remain automated when they occur within previously approved conditions. This balances continuous oversight with the practical need to retain the efficiency of agent-assisted analysis.

## 8. Illustrative Bioinformatics Cases: When Runnable Analyses Do Not Merit Trust

Technically successful analyses may still produce unreliable conclusions when defects occur in study design, statistical inference, or biological interpretation. The following hypothetical cases are provided solely as illustrative scenarios showing how these failures may arise in single-cell transcriptomics, microbial genomics, and cancer genomics. They are not reported failures of the published agentic systems discussed elsewhere in this review. Each case begins with an apparently successful workflow but contains defects that should prevent or restrict the final claim.

The five validation gates provide a structured method for identifying these defects. The execution gate evaluates computational integrity, whereas the design and inference gates assess whether the data and statistical analysis can support the intended comparison. The biological and external gates determine whether the interpretation is supported by appropriate controls, independent knowledge, and evidence beyond the originating analysis. The decision-rights profile and claim-to-evidence graph further identify which automated choices affected the result and whether the route to the final conclusion can be reconstructed.

### 8.1. Single-Cell RNA-Sequencing Analysis

Consider an agent asked to identify a disease-associated immune-cell state from a processed single-cell RNA-sequencing dataset. The available inputs include an expression matrix, donor labels, batch information, and a brief description of the study. The agent performs quality control, integrates the datasets, selects a clustering resolution, annotates the clusters, tests differential expression, and conducts pathway-enrichment analysis. All commands execute successfully, the marker plots appear plausible, and an inflammatory pathway is enriched. However, disease status is completely confounded with sequencing batch, and individual cells are analysed as independent replicates. The agent nevertheless reports a disease-specific immune-cell state.

The execution gate may pass because the data object, code, and outputs are technically valid. The design gate fails because the effects of disease and sequencing batch cannot be separated. The inference gate also fails because cell-level testing ignores the dependence among cells obtained from the same donor. The biological gate may identify additional concerns, such as incompatible lineage markers, doublet signatures, or weak consistency with reference cell types. Single-cell integration can reduce technical variation but may also remove genuine biological differences when applied inappropriately [34]. Doublet detection is necessary because mixed-cell droplets can generate artificial intermediate populations [32]. Differential analysis in multi-sample studies must preserve donor-level replication rather than treating each cell as an independent experimental unit [33]. Benchmarking also demonstrates that the validity of differential-expression results depends strongly on the analytical unit and study design [67].

The appropriate response is not to continue testing alternative clustering resolutions until a clearer disease-associated population appears. The system should abstain from the disease-specific claim and report that the design does not allow disease and batch effects to be distinguished. The provenance record should retain the donor and batch manifests, filtering thresholds, integration method, clustering resolutions explored, marker statistics, annotation evidence, and failed validation gates. CASSIA [21] demonstrates how annotation decisions and evidence can be preserved across specialized agent roles. Language-model consensus approaches may contribute to cell-type annotation, but agreement among models does not replace marker-level and donor-level validation [78]. Foundation-model performance must similarly be distinguished from the validity of downstream biological inference [41]. Reference-free annotation approaches can provide complementary evidence but do not resolve confounded study designs [79].

### 8.2. Microbial Genomics and Antimicrobial Resistance

Consider an agent asked whether two hospital isolates represent recent transmission and whether they carry clinically important antimicrobial resistance. The system downloads genome assemblies, confirms the reported species, assigns lineages, detects resistance determinants, constructs a phylogeny, and calculates genomic relatedness. The workflow completes successfully. However, one assembly is contaminated, the isolates were generated using different sequencing and assembly procedures, and the selected reference genome is genetically distant from one lineage. The agent detects a resistance gene and a short genomic distance, then reports probable ward transmission and phenotypic resistance.

Several distinct claims have been improperly combined. Assembly quality and taxonomic assignment affect whether the genomes can be compared reliably. Resistance-gene detection supports a genotypic prediction but does not automatically establish phenotypic resistance. Genomic proximity indicates relatedness but does not independently demonstrate direct transmission. Standardized microbial-genomics workflows can improve assembly reproducibility and quality assessment [80]. Long-read consensus approaches can reduce assembly error but still require contamination and completeness checks [81]. Provenance records are required to reconstruct differences in sequencing, assembly, and downstream processing [55]. ResFinder supports the detection and interpretation of acquired resistance genes and chromosomal mutations [82]. Curated antimicrobial-resistance databases strengthen genotype interpretation but remain dependent on organism, allele, and phenotype context [83]. AMRColab [84] demonstrates how standardized resistance detection can be presented transparently without treating a genomic prediction as a confirmed phenotype.

The design gate requires compatible sampling dates, locations, epidemiological metadata, and a clearly defined transmission question. The inference gate evaluates reference bias, recombination, genomic-distance thresholds, and uncertainty arising from heterogeneous sequencing procedures. The biological gate compares genotypic resistance predictions with antimicrobial-susceptibility results when available. The external gate may require epidemiological linkage, contact information, environmental sampling, or additional isolates. Genomic studies of tuberculosis show that transmission inference depends on population structure and sampling context [85]. Hospital genomic surveillance likewise requires epidemiological information to interpret genomic relatedness [86]. Similar limitations apply to the reconstruction of transmission involving syphilis and *Clostridioides difficile* [87,88]. The final decision record should therefore separate the detected resistance determinant, predicted phenotype, genomic distance, cluster assignment, and epidemiological conclusion. Viral Sentry AI [23] illustrates why downstream actions initiated by genomic risk scores require explicit authority and human approval. BIOGEN [22] demonstrates how evidence identifiers and evidence tiers can support microbial interpretation while retaining uncertainty and limitations.

### 8.3. Human Variant and Cancer Genomics

Consider an agent asked to identify clinically important variants in a cancer cohort. The agent combines variant files from different releases, annotates the variants using public databases, stratifies patients into molecular subgroups, fits survival models, and generates treatment-oriented interpretations. The analysis executes successfully, but the dataset contains mixed genome builds, tumour purity is not considered, ancestry is unevenly represented, and patient subgroups are selected after inspection of the survival curves. A recurrent variant is then described as a therapeutic biomarker despite uncertain oncogenicity and the absence of an independent validation cohort.

Cancer-genomics platforms facilitate the exploration of molecular and clinical data but do not remove the need for rigorous interpretation. cBioPortal [89] supports integrated exploration of cancer genomic and clinical datasets. GEPIA [90] enables expression-based comparisons using tumour and normal tissues. Longitudinal GENIE resources support analysis of genomic alterations and clinical outcomes across real-world oncology cohorts [91]. Variant allele frequency may provide information about clonality but is influenced by tumour purity, copy-number state, and sample composition [92]. Somatic variant-classification frameworks distinguish evidence for oncogenicity, clinical significance, and therapeutic actionability [93]. Best-practice recommendations further require disease-specific evidence categories, transparent reasoning, and appropriate qualification of uncertain findings [94,95,96]. Directly translating a database annotation into treatment language bypasses these distinctions.

The design gate requires harmonized genome builds, a clearly defined cohort, appropriate ancestry and tumour-context information, and prospectively specified comparisons. The inference gate should reject post hoc survival analyses that lack multiplicity correction or independent validation. The biological gate must distinguish pathogenicity, oncogenicity, predictive relevance, therapeutic actionability, and clinical utility. The external gate requires evidence proportional to the strength of the claim, such as an independent cohort, orthogonal assay, functional study, prospective evaluation, or established clinical evidence. Liquid-biopsy research illustrates the potential of genomic monitoring while emphasizing the need for analytical and clinical validation [97]. Precision-oncology applications similarly require careful separation of technical detection from clinical actionability [8]. AI-HOPE-WNT [24] demonstrates that an agent-assisted workflow can reproduce previously reported cancer-genomic findings. Such recapitulation supports analytical credibility but does not independently establish a new therapeutic biomarker or clinical recommendation.

Across all three cases, the primary defects occur beyond command execution. The single-cell case fails because the study design and statistical unit do not support a disease-specific claim. The microbial-genomics case fails because genomic observations are converted into phenotypic and epidemiological conclusions without sufficient contextual evidence. The cancer-genomics case fails because heterogeneous data, post hoc analysis, and uncertain variant interpretation are translated into therapeutic language. These hypothetical examples demonstrate how validation gates can identify when an analysis should stop or be downgraded, how decision-rights records can expose consequential automated choices, and how claim-to-evidence links can preserve the information required to challenge a biological conclusion.

## 9. THREAD-Bio: A Proposed Framework for Reporting Biological Evidence in Agentic Bioinformatics

Trustworthy agentic bioinformatics requires more than a record of commands, software versions, and generated outputs. An agent may reinterpret an objective, retrieve changing external resources, revise its analytical plan, retry failed operations, and formulate biological conclusions within the same analytical episode. A reporting framework must therefore capture not only what the system executed but also which decisions it made, what evidence it used, how the results were validated, and who approved consequential actions.

The Traceable History of Research Evidence, Agent Actions, and Decisions in Bioinformatics, or THREAD-Bio, is proposed as a compatibility framework for connecting these elements. It is intended to complement established standards rather than replace them. THREAD-Bio organizes the information needed to reconstruct how biological questions, data, computational actions, analytical decisions, evidence, validation outcomes, and human approvals contributed to a final claim. It should therefore be regarded as a proposed reporting structure requiring prospective evaluation and refinement rather than as an established reporting standard.

### 9.1. From Existing Standards to an Agent-Specific Reporting Framework

The FAIR principles [46] provide a foundation for making research objects findable, accessible, interoperable, and reusable. CWLProv [47] represents the provenance of computational workflow entities, activities, and agents. BioCompute Objects [48] provide structured descriptions of computational analyses, including inputs, execution environments, error handling, and outputs. Biomedical ontologies and minimum-information frameworks further support standardized descriptions of samples, assays, and experimental variables [98,99]. Medical artificial-intelligence reporting initiatives address model development, data, evaluation, and intended use [100,101].

These resources provide important foundations for agentic bioinformatics, but they do not consistently record how agent-mediated decisions allow a computational observation to become a biological claim. This gap becomes important when a system can change a dataset, substitute a reference resource, revise an analytical parameter, retrieve new evidence, or alter the interpretation after examining intermediate results. Model identity, prompt text, and execution logs alone cannot reconstruct the biological consequences of these decisions.

THREAD-Bio therefore adds a connected decision and claim layer to existing provenance structures. Its ten domains cover the biological question and study design; data and reference provenance; agent specification; instructions and decision rights; tool execution; analytical validation; biological validation; human oversight; reproducibility materials; and ethics and governance. These domains connect donor identities, filtering choices, marker evidence, and cell annotations in single-cell studies. They connect isolates, assemblies, resistance databases, genomic relatedness, and epidemiological interpretation in microbial genomics. They also connect genome builds, tumour characteristics, variant evidence, and clinical wording in cancer genomics.

### 9.2. How THREAD-Bio Extends Current Reporting Practices

Published agentic bioinformatics systems already provide several components required by THREAD-Bio. Biological objectives, model or agent architectures, input data, computational tools, execution processes, evaluation procedures, and reproducibility resources are often described to some degree. Open software, benchmark datasets, computational notebooks, source identifiers, interaction records, and expert evaluations provide useful foundations. The purpose of THREAD-Bio is not to duplicate these materials but to connect them to individual decisions, validation states, evidence records, and biological claims.

The reporting objects within the framework serve different purposes. An execution record documents commands, tools, retries, and generated artifacts. A decision record explains how one option was selected among alternatives. An evidence record identifies supporting, indirect, contradictory, or outdated information. A validation record identifies the gate applied, the evidence examined, and whether the analysis passed, failed, or proceeded with restricted claims. A human-approval record identifies the responsible reviewer, the consequential decision being reviewed, and whether that decision was approved, rejected, or overridden. These records do not require disclosure of private model reasoning. Concise summaries of consequential decisions are sufficient for scientific inspection. Table 4 translates the ten THREAD-Bio domains into a concise set of reporting requirements. The structure is intended to help authors, reviewers, developers, and institutions determine whether the route from a biological question to a final claim can be reconstructed.

### 9.3. Reporting Gaps, Feasibility, and Required Validation

The least consistently described aspects of the published systems were those most specific to autonomous scientific decision-making. Information on agent architecture, prompts, analytical roles, and execution processes was often available, but permission boundaries, conditions for human approval, reversibility, scientific consequences, and data sensitivity were not consistently reported in a form that allowed direct comparison across systems.

Biological validation, human oversight, and ethics and governance were also described with varying levels of detail. Some publications reported expert assessment, restricted execution, local deployment, sensitive-data handling, evidence records, or other relevant safeguards, whereas others focused primarily on technical performance or workflow execution. Importantly, incomplete reporting does not demonstrate that a safeguard was absent from the underlying system or its deployment environment. It indicates only that the information could not be reconstructed reliably from the published report.

THREAD-Bio remains a proposal rather than a consensus reporting standard. Its terminology, domains, and reporting burden require independent evaluation. Multiple researchers should apply the framework to the same systems and measure agreement, completion time, ambiguous items, and information that cannot be recovered from published reports. Prospective studies should test whether THREAD-Bio records expose errors that ordinary workflow logs miss, improve reviewer assessment, or support reconstruction of consequential decisions.

Reporting burden is a legitimate concern. Preserving every token, intermediate state, or routine command would create unreadable records and may introduce privacy and storage risks. Event-based reporting provides a more practical approach. Routine operations can be summarized automatically, while explicit records are retained when an agent excludes a sample, changes a reference genome, modifies a primary contrast, adjusts a consequential threshold, or revises a mechanistic or clinical claim. CWLProv [47] and BioCompute Objects [48] may populate many computational fields automatically. Human authors can then concentrate on the biological decisions and justifications that cannot be inferred from workflow lineage alone.

The framework should be revised or simplified if independent reviewers cannot distinguish its domains or if the burden of implementation exceeds its scientific value. Validation gates are useful only if they identify invalid analyses more effectively than existing review practices. Claim-to-evidence graphs are useful only if they improve reconstruction, error detection, disagreement resolution, or calibration of biological claims. THREAD-Bio should therefore be treated as a testable hypothesis: accountability may become easier to evaluate when agent actions, decisions, evidence, validation outcomes, human approvals, and claims are preserved as one connected scientific record.

## 10. Benchmarking Biological Validity and Research Priorities

Most benchmarks for computational systems emphasize task completion, executable code, agreement with a reference answer, or similarity to an established workflow. These measures are useful for evaluating technical performance, but they do not determine whether an analysis is scientifically appropriate or whether its biological conclusion is adequately supported. An agent may reproduce a reference workflow while overlooking a fatal defect in study design, statistical inference, or interpretation.

Benchmarks for agentic bioinformatics should therefore evaluate whether a system recognizes when a technically runnable analysis does not merit a strong biological claim. Relevant outcomes include detection of scientific defects, appropriate abstention, calibration of conclusions, completeness of the analytical trace, and preservation of decision context after replanning. Prospective evaluation should also determine whether these safeguards improve scientific review without creating disproportionate reporting or computational burdens.

### 10.1. From Task Completion to Biological Validity

Biological-validity benchmarks should include tasks in which data files are readable and commands can execute successfully, but the requested analysis remains scientifically inappropriate. Examples include differential-expression analysis based on pseudoreplicated observations, paired samples analysed as independent groups, complete confounding between phenotype and batch, post hoc contrast selection, mixed genome builds, leakage between training and validation data, and pathway enrichment using an unsuitable background set. The central evaluation criterion is whether the agent identifies the defect and restricts the resulting claim rather than merely producing an output.

Illustrative benchmark scenarios could include a single-cell dataset in which disease status is completely confounded with sequencing batch, requiring the system to withhold a disease-specific interpretation; a microbial-genomics task in which contaminated or technically heterogeneous assemblies make a transmission claim unreliable; and a cancer-genomics task in which mixed genome builds, post hoc subgroup selection, and absent external validation should prevent a therapeutic claim. In each case, successful execution alone would not constitute benchmark success.

Existing omics benchmarks provide suitable foundations for constructing such tasks. Comparative studies of single-cell multimodal integration, spatial-omics analysis, normalization, imputation, and differential-expression workflows provide datasets with known analytical sensitivities [67,68,69,102,103]. Microbiome workflow benchmarking similarly demonstrates that preprocessing, classification, and analytical choices can materially alter downstream findings [104]. Agentic benchmarks can introduce controlled design, inference, or interpretation defects into these established datasets rather than relying only on general question-answering tasks.

Benchmark design must also preserve construct validity. A task should measure whether an agent identifies a scientific problem, not whether it reproduces the benchmark developer’s preferred software or pipeline. Several analytical approaches may be defensible, and abstention may be more appropriate than completing the requested workflow. Scoring should therefore separate defect recognition, justification, safe action, trace completeness, and calibration of the final claim. A technically correct workflow should receive a low score when it ignores a design flaw that invalidates the biological conclusion.

Evaluation metrics should likewise be reported separately rather than collapsed into a single performance score. Useful measures include the proportion of predefined scientific defects correctly identified, the frequency of inappropriate continuation despite a critical defect, appropriate abstention, agreement between the strength of the final claim and the available evidence, completeness of claim-to-evidence links, reproducibility of the resulting analysis, reviewer workload, and additional computational cost. The acceptable level for each metric should depend on the task and intended use rather than on a universal threshold.

### 10.2. Measuring Abstention, Calibration, and Trace Completeness

Abstention benchmarks should vary the amount and quality of available information. Some tasks may be fully answerable, whereas others may require additional metadata, clarification of the study design, or restriction to descriptive analysis. The agent should identify what information is missing, explain how the omission affects inference, and indicate whether a limited analysis remains possible. A system should not infer critical design information from filenames, sample labels, or contextual assumptions merely to complete the workflow.

Calibration should be assessed against defined evidence states rather than a generic confidence score. Outputs may be classified as technically verified, statistically supported, biologically plausible, externally replicated, or experimentally validated. The wording of the conclusion should remain proportional to the highest evidence state achieved. A pathway association should not be presented as a mechanism, an internally validated classifier should not be described as clinically useful, and genomic relatedness should not be translated directly into confirmed transmission.

Trace completeness can be measured using coverage of the claim-to-evidence graph. Evaluators can determine whether a final claim remains linked to the input samples, reference resources, consequential decisions, computational actions, statistical results, supporting evidence, validation outcomes, and human-approval states where applicable. Correct and interpretable links are more important than the volume of retained logs. Adversarial tests may remove metadata, alter reference versions, introduce contradictory controls, or insert misleading documentation. Related examples show how apparently reasonable analytical outputs can exceed the evidence available for rhythmic programmes, microbial functions, or biological interpretability [105,106,107].

### 10.3. Prospective Evaluation and Community Development

Prospective studies should evaluate agents within complete research projects rather than through isolated prompts. Fixed workflows, conversational copilots, and autonomous agents could be compared using the same biological questions and datasets. Outcomes should include completion time, analytical errors, reversibility of decisions, reviewer workload, abstention behaviour, reproducibility, and calibration of final claims. Independent bioinformaticians should assess whether the computational analyses are methodologically defensible, while biological experts should evaluate whether the interpretations are useful and proportionate to the evidence.

A practical first step could be a small prospective pilot in which the same predefined bioinformatics tasks are completed using three conditions: a fixed researcher-defined workflow, an AI copilot that provides recommendations without independently changing the analytical route, and an autonomous agent permitted to plan and revise parts of the analysis. The comparison should use the same input data, biological question, predefined scientific defects, and evaluation criteria. Such a study would test whether increasing autonomy improves efficiency without increasing unsupported analytical decisions or biological claims.

The proposed failure categories, decision-rights profile, validation gates, and THREAD-Bio framework also require reliability testing. Independent researchers should apply each component to the same publications and analyses, with agreement, ambiguous classifications, completion time, and unresolved information reported. Prospective incident repositories could classify failures as inherited, amplified, or emergent and determine whether these categories predict scientific consequence, detectability, or remediation difficulty. Poor agreement would indicate that the terminology or decision boundaries require revision.

Community development of these benchmarks should involve bioinformaticians, biological domain experts, statisticians, software developers, and users of agentic systems. Shared benchmark sets should use versioned datasets, clearly documented scientific defects, predefined expected responses, and independent expert adjudication. Hidden or periodically refreshed test cases would help reduce adaptation to a fixed benchmark, while multi-centre evaluation could determine whether results generalize across research environments and biological domains.

Finally, research should determine whether improved traceability changes scientific outcomes. A complete record is valuable only if it helps investigators reproduce analyses, identify invalid decisions, resolve disagreements, calibrate claims, or prevent unsupported conclusions. Reviewer studies could compare conventional notebooks with structured claim-to-evidence records. Developers could measure the additional storage, computation, and labour required to generate these records. Institutions could evaluate consequence-sensitive human-approval policies using public, restricted, and clinical datasets. Until such evidence is available, accountable autonomy should be regarded as an active research programme rather than an established solution.

## 11. Conclusions

Agentic artificial intelligence can broaden access to genomic, transcriptomic, and other bioinformatics analyses by supporting tool selection, code execution, iterative planning, and biological interpretation. However, greater analytical autonomy does not necessarily produce stronger scientific evidence. The main concern is not simply that agents may make errors, because conventional bioinformatics workflows already contain risks involving data quality, reference selection, statistical modelling, and interpretation. The distinctive challenge is that agentic systems can rapidly propagate, revise, and compress the analytical path through which these errors become persuasive biological claims.

Current systems already implement valuable safeguards, including restricted execution environments, activity logs, evidence identifiers, automated checks, critic agents, quality scores, and expert assessment. These measures should be retained and strengthened. However, the published reports examined in this review often describe these safeguards separately and do not consistently provide a connected record linking the biological question, input data, analytical decisions, computational actions, statistical results, supporting evidence, validation outcomes, and human approvals. The proposed failure framework, decision-rights profile, validation gates, claim-to-evidence architecture, and THREAD-Bio reporting domains are intended to make this route more transparent and scientifically inspectable.

These proposals remain preliminary. THREAD-Bio is not a consensus reporting standard, and complete documentation cannot by itself establish biological validity. The framework requires independent assessment, prospective evaluation, reliability testing, and simplification where its reporting burden exceeds its practical value. Trustworthy agentic bioinformatics will ultimately depend not only on whether an agent can complete an analysis, but also on whether its decisions and conclusions can be reconstructed, challenged, appropriately validated, and held accountable under responsible scientific oversight.

## Figures and Tables

**Table 1:** Descriptive characteristics and reported validation, evidence, and oversight features of selected PubMed-indexed agentic bioinformatics systems.

System	Biological Application	Reported Agentic Role	Reported Validation/Evidence Approach	Human Oversight	Reference
AutoBA	Multi-omics workflow analysis	Planning, code generation, execution, and automated repair	Sandboxed execution, restricted commands, documented failures, and review of generated outputs	Bioinformatician review reported	[15]
BRAD	Biomarker discovery and evidence retrieval	Literature retrieval, database access, analytical planning, and tool use	Interaction and decision logs linked to cited literature	Not clearly described as a formal approval step	[16]
BioAgents	Genomics support and workflow recommendation	Provides genomics guidance and analytical recommendations	Comparison with bioinformatics experts	Expert involvement reported; formal approval conditions not specified	[17]
BioMedAgent	Cross-omics, machine-learning, and biomedical-imaging analysis	Coordinates multiple tools and analytical steps	Benchmark tasks, reference steps, analytical milestones, and external evaluation	Formal human approval requirements not clearly reported	[18]
CellVoyager	Single-cell RNA-sequencing analysis	Iterative exploration and analysis in computational notebooks	Expert assessment across case studies	Expert assessment reported	[1]
Agentomics	Biomedical machine-learning analysis	Iterative model development and analytical experimentation	Restricted environment, hidden test labels, deterministic metrics, and artifact validation	Programmatic checkpoints reported	[19]
BIOGEN	Bacterial transcriptomic interpretation	Evidence retrieval and multi-critic biological interpretation	Source identifiers, evidence tiers, critic assessments, and stated limitations	Formal human approval not clearly reported	[22]
Viral Sentry AI	Viral surveillance and host-risk prediction	Links genomic surveillance, risk prediction, and downstream analysis	Cross-validation, threshold-based triggering, and manual hallucination assessment	Manual assessment reported	[23]
AI-HOPE-PM	Precision-medicine analysis	Integrates genomic, clinical, and social information	Query-parsing benchmarks and executable statistical workflows	Formal translational approval not reported	[25]
AI-HOPE-WNT	Colorectal cancer genomic analysis	Conversational genomic analysis and interpretation	Reproduction of previously reported genomic findings	Formal approval process not clearly reported	[24]
CASSIA	Single-cell cell-type annotation	Multi-agent annotation, validation, scoring, and reporting	Recorded conversations, validation decisions, supporting evidence, and quality scores	Mandatory expert approval not clearly reported	[21]

**Table 2:** Proposed decision-rights profile for agentic bioinformatics.

Decision Domain	Representative Delegated Action	Principal Scientific or Operational Risk	Minimum Evidence to Retain
Data	Selection of datasets, samples, comparison groups, or exclusion criteria	Redefinition of the study population, introduction of selection bias, or exposure of sensitive information	Eligibility criteria, sample manifest, inclusion and exclusion records, rationale, and responsible approver
Methods	Selection of workflows, software, genome references, annotation resources, or analytical strategies	Use of an incompatible or unsuitable method that changes the biological interpretation	Alternatives considered, selection rationale, compatibility checks, software versions, and reference-resource versions
Parameters	Selection or modification of thresholds, defaults, filters, clustering resolutions, or database releases	Alteration of detected features, excluded observations, or reported discoveries	Parameter values, justification, default source, sensitivity analyses, and override history
Statistics	Definition of the estimand, contrast, covariates, dependence structure, multiplicity procedure, or statistical model	Invalid inference, confounding, pseudoreplication, data leakage, or unsupported confirmatory claims	Analysis specification, model assumptions, diagnostics, multiplicity plan, sensitivity analyses, and approver
Execution	Running commands, retrying failed operations, installing software, overwriting files, or accessing external resources	Irreversible modification, unauthorized access, undocumented repair, or propagation of computational errors	Commands, computing environment, accessible resources, retry history, repair actions, integrity checks, and generated artifacts
Interpretation	Assignment of cell identities, pathways, mechanisms, resistance phenotypes, causal meaning, or clinical relevance	Overstatement of association, unsupported mechanistic inference, or inappropriate biological or clinical action	Claim-to-evidence links, supporting and contradictory evidence, uncertainty, evidence state, and responsible reviewer
Reporting	Generation of figures, summaries, conclusions, or manuscript text	Selective reporting, exaggeration of evidence, loss of uncertainty, or disclosure of protected information	Draft history, source and evidence links, disclosure of agent contributions, evidence status, and author approval

**Table 3:** Proposed validation gates for agentic bioinformatics analyses and biological claims.

Validation Gate	Central Criterion	Minimum Evidence Required	Failure or Abstention Condition	Human Approval Requirement
Execution	Correct completion of the computational operation	Valid commands and logs, expected output files, correct schemas, non-empty results, plausible values, and documented retries	Missing or corrupted outputs, integrity failures, incompatible formats, or undocumented repair attempts	Human review when automated checks fail, produce ambiguous results, or are unavailable
Design	Suitability of the analysis for addressing the biological question	Defined cohort, sample manifest, independent experimental units, comparison groups, covariates, metadata completeness, and compatible reference resources	Incompatible cohort, perfect confounding, absent biological replication, unclear comparison groups, or missing essential metadata	Approval of the final cohort, exclusion criteria, reference resources, and principal comparison
Inference	Defensibility of statistical assumptions, uncertainty estimates, and inferential claims	Prespecified model, estimand, contrast, covariates, dependence structure, multiplicity procedure, diagnostics, and sensitivity analyses	Data leakage, pseudoreplication, invalid model assumptions, unstable results, or exploratory analyses presented as confirmatory	Review and approval of principal statistical analyses and inferential claims
Biological	Biological support for the proposed interpretation	Appropriate controls, coherent markers or phenotypes, contradiction searches, evidence labels, and domain-specific biological assessment	Failed controls, incompatible biological evidence, unresolved contradictions, or mechanistic claims based only on plausibility	Domain-expert approval for mechanistic, causal, translational, or clinically relevant interpretations
External	Evidence extending beyond the originating analysis	Independent cohort, orthogonal analytical platform, functional assay, perturbation experiment, prospective evaluation, or clinical replication	Absence of relevant external support, unsuccessful replication, or evidence insufficient for the strength of the claim	Explicit approval before making strong causal, translational, or clinical claims

**Table 4:** Proposed THREAD-Bio reporting domains for agentic bioinformatics.

Code	THREAD-Bio Domain	Minimum Information to Report
A	Biological question and study design	Research question, population, comparison, experimental units, and biological constraints
B	Data and reference provenance	Samples, metadata, reference resources, versions, and agent-mediated changes
C	Agent specification	Model or system, version, analytical roles, memory use, and coordination structure
D	Instructions and decision rights	Permitted, restricted, autonomous, and human-approved actions across the analytical workflow
E	Tool execution	Tools, commands, environments, retries, failures, substitutions, and generated outputs
F	Analytical validation	Execution, design, and inference checks, including failures and abstention decisions
G	Biological validation	Biological evidence, controls, contradictory evidence, external support, and limits of interpretation
H	Human oversight	Responsible reviewers, decisions requiring human approval, approvals, rejections, and overrides
I	Reproducibility package	Data, code, parameters, environments, workflow records, and relevant agent-action records
J	Ethics and governance	Privacy, security, data use, access permissions, sensitive-data handling, and disclosure of agent contributions

## Data Availability

No new data were created or analyzed in this study. Data sharing is not applicable to this article.

## Supplementary Materials

The following supporting information can be downloaded at https://www.mdpi.com/article/10.3390/biology15171537/s1: Table S1: Descriptive characteristics of agentic bioinformatics systems included in the focused review [1,15,16,17,18,19,21,22,23,24,25].
