# Supplementary material for: Trustworthy Agentic AI in Bioinformatics: From Workflow Automation to Traceable and Validated Biological Inference
Source: Biology (Basel). 2026 Sep 4;15(17):1537. doi: 10.3390/biology15171537 (PMC13565537; doi:10.3390/biology15171537)
Supplement: Supplementary file 1 [file biology-15-01537-s001.zip › biology-4510575-supplementary.pdf]

Supplementary Table S1. Descriptive characteristics of agentic bioinformatics systems included in the focused review.

System	Biological application / data modality	Model / agent architecture	Reported autonomy / analytical role	Reported validation / evidence approach	Human oversight	Provenance / reporting features	Code / data availability
AutoBA [15]	Multi-omics bioinformatics; sequencing-based omics datasets	LLM-based autonomous agent with multiple LLM backends and automated code-repair mechanism	Generates analytical plans, produces and executes code, performs analyses, and repairs failed operations	Tested across real-world multi-omics analysis scenarios; sandboxed/restricted execution, documented failures, and review of generated plans and outputs	Bioinformatician review reported	Stepwise analytical plans, failed operations, and repair cycles are retained/reported	Open-source implementation; online and local deployment supported
BRAD [16]	Biomarker discovery and enrichment; RNA-seq data, scientific literature, and biological databases	LLM-based modular agent built around planning, memory, RAG/document retrieval, database access, and software tools	Selects and executes analytical software, retrieves evidence, performs biomarker/enrichment analysis, and generates reports	Retrieval and enrichment outputs evaluated against reference information; literature citations provide verifiable evidence	Human review/refinement reported in the demonstrated workflow; human approval gate not specified	Detailed JSON interaction and decision logs, source records, intermediate outputs, and reproducibility reports	Source code publicly available
BioAgents [17]	Genomics and bioinformatics workflow support	Multi-agent system using fine-tuned small language models with retrieval-augmented generation	Provides genomics guidance, workflow recommendations, and code/methodological assistance	Outputs compared with bioinformatics experts	Expert involvement reported; formal approval conditions not specified	Detailed claim-level provenance not clearly reported in the focused extraction	Source code publicly available; study datasets available from authors on request
BioMedAgent [18]	Cross-omics, biomedical machine learning, and biomedical imaging	Self-evolving LLM-based multi-agent framework with tool-aware specialized agents and automated evaluation	Plans tasks, selects and chains tools, executes analyses, and summarizes results	BioMed-AQA benchmark using reference steps and analytical milestones, with additional external evaluation	Formal human approval requirements not clearly reported	Planning, execution, milestones, and task summaries can be retained and inspected	Open-source code; benchmark questions, reference milestones, and associated data publicly available
CellVoyager [1]	Single-cell RNA-sequencing	LLM-based autonomous computational-biology agent	Autonomously proposes hypotheses, selects subsequent analyses, implements	CellBench benchmark and expert assessment	Expert assessment reported; researchers may interact with the	Executable notebook-based analytical record preserves	Code and CellBench resources publicly available through GitHub/Zenodo;

		operating within Jupyter notebooks	analyses, and interprets intermediate findings	of biological case-study outputs	notebook-based analysis	generated analyses and intermediate outputs	case-study datasets traceable to original studies
Agentomics [19]	Biomedical machine learning; regulatory genomics, protein engineering, and drug discovery	Autonomous LLM-powered agentic system for end-to-end ML experimentation	Iteratively develops, evaluates, and revises ML strategies and produces trained models and reusable artifacts	Restricted execution environment, strict validation checkpoints, hidden test labels, deterministic metrics, and artifact validation	Programmatic checkpoints reported; formal human approval not required during routine experimentation	Validated code, model artifacts, interfaces, and analytical outputs are retained	Open-source Python implementation and documentation publicly available
BIOGEN [22]	Bacterial transcriptomic interpretation, particularly antimicrobial-resistance-associated RNA-seq modules	Evidence-grounded multi-agent framework combining biomedical retrieval, structured interpretation, and multiple critic agents	Retrieves and synthesizes external evidence, generates biological interpretations, and subjects them to critic evaluation	Identifier verification, evidence tiers, critic assessments, semantic/functional evaluation, ablation analyses, and evaluation under distribution shift	Formal human approval not clearly reported	Source identifiers, intermediate evidence, evidence tiers, critic assessments, and stated limitations	Study materials are provided in the article/supplementary material; a dedicated public code repository was not clearly identified in the publication
Viral Sentry AI [23]	Viral-genome surveillance, host-risk prediction, and drug-repurposing analysis	Agentic analytical pipeline; detailed underlying model architecture not clearly described in the reviewed manuscript information	Connects viral surveillance and host-risk prediction with threshold-triggered downstream drug-repurposing analysis	Cross-validation, threshold-based triggering, and manual hallucination assessment	Manual assessment reported; formal human approval conditions not clearly reported	Detailed claim-level provenance not clearly reported	Not clearly reported in the reviewed information
AI-HOPE-WNT [24]	Colorectal-cancer precision oncology; clinical and genomic WNT-pathway data	Conversational AI agent for natural-language-driven clinical-genomic analysis	Interprets user queries and automates pathway-focused genomic, statistical, and clinical analyses	Demonstrated by reproducing previously reported colorectal-cancer genomic findings	Formal approval process not clearly reported	Detailed claim-level provenance not clearly reported	Code/data availability not clearly described in the information reviewed for this extraction
AI-HOPE-PM [25]	Precision medicine;	Conversational RAG framework	Converts natural-language questions	Query-parsing benchmark,	Clarification prompts support	Reproducible Python code	Platform, source code, demonstration

	genomic, clinical, treatment, demographic, and social-determinant data	using a fine-tuned biomedical LLaMA 3 model with Python analytical pipelines	into cohort selection, case-control analyses, survival analyses, odds-ratio testing, visualization, and interpretation	comparison with established/manual analyses, and evaluation of analytical fidelity	user interaction; human approval gate for translational use not reported	logs can be retained and exported	datasets, example queries, and documentation publicly available
CASSIA [21]	Single-cell RNA-sequencing cell-type annotation	Multi-agent LLM framework with interconnected annotation, validation, formatting, quality-scoring, and reporting agents; optional RAG	Performs iterative annotation, validation, quality scoring, revision, and reporting	Benchmarking across multiple datasets/cell types, internal validation, quality scoring, mixed-population testing, and repeated-model evaluation	Mandatory expert approval not clearly reported	Conversation histories, validation decisions, supporting evidence, quality scores, and failure reports are retained	Code publicly available through GitHub/Zenodo; generated and processed scRNA-seq data and supplementary datasets available

Abbreviations: LLM, large language model; RAG, retrieval-augmented generation; ML, machine learning; RNA-seq, RNA sequencing; scRNA-seq, single-cell RNA sequencing. “Not clearly reported” indicates that the corresponding information could not be verified from the publication or supporting material reviewed and should not be interpreted as evidence that the feature was absent.
